# Environmental drivers of crocodyliform extinction across the Jurassic/Cretaceous transition

**DOI:** 10.1098/rspb.2015.2840

**Published:** 2016-03-16

**Authors:** Jonathan P. Tennant, Philip D. Mannion, Paul Upchurch

**Affiliations:** 1 Department of Earth Science and Engineering, Imperial College London, London SW6 2AZ, UK; 2 Department of Earth Sciences, University College London, Gower Street, London WC1E 6BT, UK

**Keywords:** Crocodylomorpha, Neosuchia, Notosuchia, Thalattosuchia, shareholder quorum subsampling, phylogenetic diversity estimate

## Abstract

Crocodyliforms have a much richer evolutionary history than represented by their extant descendants, including several independent marine and terrestrial radiations during the Mesozoic. However, heterogeneous sampling of their fossil record has obscured their macroevolutionary dynamics, and obfuscated attempts to reconcile external drivers of these patterns. Here, we present a comprehensive analysis of crocodyliform biodiversity through the Jurassic/Cretaceous (J/K) transition using subsampling and phylogenetic approaches and apply maximum-likelihood methods to fit models of extrinsic variables to assess what mediated these patterns. A combination of fluctuations in sea-level and episodic perturbations to the carbon and sulfur cycles was primarily responsible for both a marine and non-marine crocodyliform biodiversity decline through the J/K boundary, primarily documented in Europe. This was tracked by high extinction rates at the boundary and suppressed origination rates throughout the Early Cretaceous. The diversification of Eusuchia and Notosuchia likely emanated from the easing of ecological pressure resulting from the biodiversity decline, which also culminated in the extinction of the marine thalattosuchians in the late Early Cretaceous. Through application of rigorous techniques for estimating biodiversity, our results demonstrate that it is possible to tease apart the complex array of controls on diversification patterns in major archosaur clades.

## 1. Introduction

Crocodyliforms are a major group of pseudosuchian archosaurs that include living crocodylians. Originating in the Late Triassic [1], they have a long and rich evolutionary history [2–5]. The Jurassic–Early Cretaceous interval records at least two independent marine radiations of diverse groups (Thalattosuchia and ‘Tethysuchia’ [6,7]), as well as a major phase of terrestrial diversification (Notosuchia [8]). It also includes the decline and eventual extinction of Thalattosuchia [9], and radiation of Eusuchia, the lineage leading to crown group Crocodylia [3].

Although some studies have documented high lineage survival of marine crocodyliforms across the Jurassic/Cretaceous (J/K) boundary (145 Ma) [9,10], others have recovered an overall decrease in their biodiversity [5,11], with evidence for a comparable decline among non-marine forms too [5]. Uncertainty characterizes the tempo of any decline as well, varying from an extinction event at the boundary [12,13], to a spatio-temporally staggered turnover [14] that might have comprised a pulsed, two-phase wave of extinctions [15]. Alternate explanations for fluctuations in marine crocodyliform biodiversity across the J/K boundary have also been proposed, including close ties to changes in sea level [5] and palaeotemperature [11], whereas the driver/s of patterns in non-marine crocodyliform biodiversity have yet to be identified for this interval. Thus, there is considerable uncertainty concerning both the patterns of biodiversity change across the J/K boundary for marine and non-marine crocodyliforms, and the identity of the causal factors that supposedly drove such fluctuations.

These disagreements are likely to, at least in part, stem from contrasting approaches to the reconstruction of palaeobiodiversity patterns. While recent analyses of crocodyliforms based on uncorrected (raw) taxonomic counts, phylogenetically corrected biodiversity and subsampling approaches [5,11] largely recover the same patterns, they differ in the magnitude of these changes and their potential driving factors. The construction of large fossil occurrence databases, combined with increasingly sophisticated approaches to ameliorate the impact of heterogeneous sampling on our reading of the fossil record (e.g. [16,17]), has shown that the biodiversity of dinosaurs [18], marine reptiles [12,14,19] and some groups of marine invertebrates [20–22], also declined across the J/K boundary. Together, these studies provide renewed evidence for a more widespread and taxonomically inclusive faunal turnover during the Late Jurassic–Early Cretaceous interval.

Here, we present a detailed analysis of Jurassic–Cretaceous crocodyliform biodiversity, focusing in particular on dynamics across the J/K boundary, a relatively neglected phase in their evolutionary history. We employ a suite of analytical approaches to reconstruct crocodyliform palaeobiodiversity, including a new supertree and a range of subsampling methods, and also calculate two different measures of extinction and origination rates. Our results allow us to quantify the magnitude of crocodyliform biodiversity fluctuations across the J/K boundary and provide insight into the environmental mechanisms that underpinned these macroevolutionary changes.

## 2. Material and methods

### (a) Occurrence dataset

**Figure 1.**
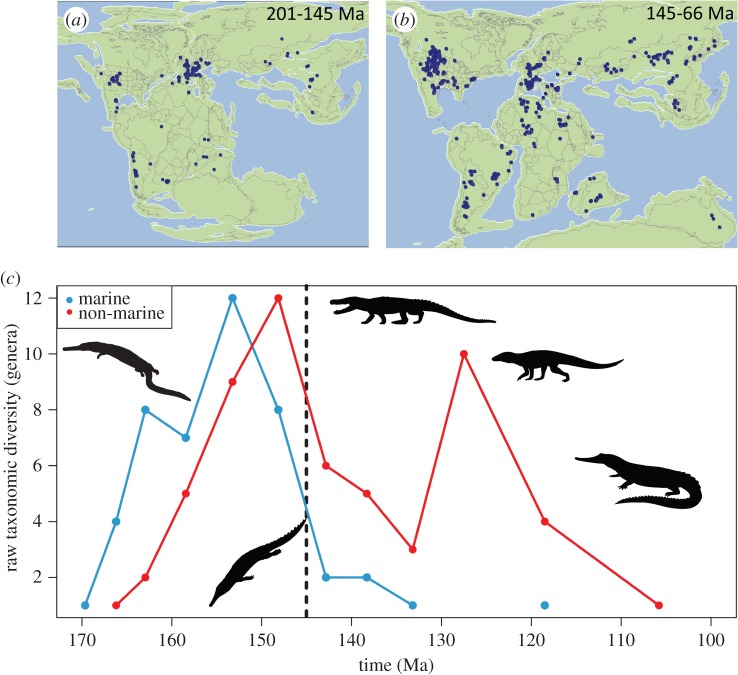
Jurassic (*a*) and Cretaceous (*b*) crocodyliform occurrences, superimposed onto reconstructed palaeomaps. Silhouettes: *Isisfordia* (M. Keesey), *Goniopholis* (S. Hartman), *Notosuchus* (N. Tamura), *Steneosaurus* (G. Monger), *Elosuchus* (M. Keesey), *Protosuchus* (M. Keesey). (*c*) Raw TDE for Jurassic–Cretaceous marine (blue) and non-marine (red) crocodyliforms. Source for palaeomaps: http://fossilworks.org/?a=mapForm.

Although the main focus of our study is on patterns during the Late Jurassic–Early Cretaceous, we used a dataset spanning the entirety of the Jurassic to Cretaceous (201–66 Ma) to increase statistical power and to detect changes in longer-term trends. We used a newly compiled fossil occurrence dataset [23], comprising a near-comprehensive record of crocodyliforms. Body fossil occurrences that could be assigned to genera were downloaded from *The Paleobiology Database* (PaleoDB; http://www.paleobiodb.org/), accessed 29 July 2015. Despite issues with supra-specific assessments of biodiversity patterns [24,25], Mesozoic crocodyliform genera and species numbers are tightly correlated through time [5], and therefore genera were used to increase sample size, via the inclusion of specifically indeterminate occurrences. Genera were subdivided into those adapted to a fully aquatic lifestyle (comprising thalattosuchians, dyrosaurids, gavialoids and some pholidosaurids) and those which were non-marine and occupied terrestrial environments (including freshwater and coastal localities) (section SI 1 in [26]). We followed Mannion *et al*. [5] by excluding spurious Mesozoic occurrences of *Crocodylus* and Cretaceous occurrences of teleosauroids. This resulted in a dataset comprising 349 marine occurrences of 31 genera from 302 collections, and 825 non-marine occurrences of 132 genera from 809 collections (section SI 2 in [26]) (figure 1*a*,*b*). To explore the impact of different binning schemes, these data were pooled into: (i) approximately equal length (approx. 10 Myr, *n* = 14) time bins; and (ii) stage-level (*n* = 23) time bins (section SI 3 in [26]). Raw in-bin counts of these genera were used to produce an uncorrected taxonomic diversity estimate (TDE) (figure 1*c*). Lastly, non-marine data were subdivided into palaeocontinents (section SI 2 in [26]) to investigate regional patterns in non-marine crocodyliform biodiversity and to test whether global patterns resulting from subsampling approaches (see below) are a product of grouping non-geographically contiguous areas. All analyses were conducted in R v. 3.0.2 [27], except where stated otherwise.

### (b) Phylogenetic diversity estimation

We built a new informal crocodyliform supertree at both the genus and species levels (section SI 3 in [26]) and used these as the basis for producing a phylogenetic diversity estimate (PDE). We tested the sensitivity of this approach by resolving polytomies in three different ways: (i) in an ‘equal’ fashion, by assigning an equal portion of time to zero-length branches available from the first directly ancestral branch of positive length [28]; (ii) by randomly resolving polytomies [29]; and (iii) by resolving polytomies under the assumption that the order of first stratigraphic appearance reflects the order of branching (note that if the first appearances of two or more unresolved taxa are identical, then they are randomly resolved) [29]. Trees were dated using taxonomic first and last occurrences extracted from the PaleoDB (section SI 1 in [26]), and time-scaled using the R functions DatePhylo() (for the ‘equal’ method) and timePaleoPhy() (for the random and ordered methods) in the packages *strap* [29] and *paleotree* [30], respectively. Subsequent to the dating procedure, each supertree was divided into two subtrees for marine (86 species comprising 31 genera) and non-marine (169 species comprising 115 genera) taxa (section SI 1 in [26]), using the drop.tip() function in the *ape* package [31]. This removed the appropriate terminal and corresponding internal branches from the original supertrees. For each subtree, we calculated phylogenetic diversity as the sum of all known occurrences plus ghost lineages for each time bin (PDEt for 10 Myr bins, and PDEs for stage bins).

### (c) Shareholder quorum subsampled biodiversity

We employed shareholder quorum subsampling (SQS) as a method for correcting palaeobiodiversity estimates, by taking into account the abundance distribution of taxa. SQS samples evenly from occurrence lists, using Good's *u* as an estimation of the ‘coverage’ of the fossil record [16,20] (see section SI 3 in [26]). SQS was applied to our marine and non-marine genus-level occurrence datasets for each time interval to provide an estimate of global subsampled taxonomic richness, using two methods (each using our two binning strategies; section SI 3 in [26]). The first of these, SQSP, was conducted using a Perl script written and provided by J. Alroy, applied at 10 Myr time intervals (SQSPt) and at the stage level (SQSPs). This version of SQS allows constraint over the number of taxonomic occurrences subsampled based on their frequency per collection [16,20,32]. In this instance, whenever a collection from a new publication was sampled from the list, subsequent collections were sampled until exactly three collections from that publication had been selected [16,33]. Singletons were excluded, and dominant taxa (those with the highest frequency of occurrences per bin) were included. We set a baseline quorum of 0.4, as this has been demonstrated to be sufficient to accurately assess changes in biodiversity [32]. We ran 1000 subsampling trials per iteration and report the mean biodiversity. The result is a representation of ‘true’ biodiversity, calculated based on relative proportions of taxa per interval.

Secondly, we employed the SQS function (v. 3.3) for R available on J. Alroy's personal website. The major difference between this and the Perl script is that there is no restriction based on the number of publications per time bin, and no correction for single large collections [16,20,32]. For all analyses, we set a baseline quorum of 0.4 and performed 100 replicates of 1000 iterations to obtain a subsampled biodiversity distribution (SQSR), conducted at 10 Myr time intervals. Whereas our constrained analysis (SQSRc) restricted our dataset to occurrences that could be assigned to a single time bin, we also tested the stability of the resulting non-marine curves by assessing the influence of retaining occurrences with uncertainty in their temporal duration from the original dataset (unconstrained analysis [SQSRu]; see section SI 3 in [26]).

### (d) Extinction and origination rates

We calculated extinction and origination rates for the global, marine and non-marine occurrence datasets. We used two different measures, three-timer (3 T [33,34]) and ‘Foote’ rates [35,36]. The 3 T extinction rate (*μ*) is a per-taxon, per-interval probabilistic measure of the rate of taxa crossing the basal boundary of a bin and continuing to its top, corrected for the fact that members of this group might be present but not sampled in the following bin (i.e. the Signor–Lipps effect [37]). The 3 T origination rate (*λ*) is essentially the inverse of this [34]. The Foote method analyses boundary-crossers and is considered to be a conservative estimate of rates, as it takes the fossil record literally (i.e. assumes perfect sampling) and ignores singletons [36], but suffers from ‘edge’ effects and back-smearing of extinction rates [33]. However, boundary-crossing methods have benefits relative to in-bin methods, in that the former ameliorates issues pertaining to the grouping of taxa within bins that might not have coexisted (i.e. some taxa might have gone extinct before others had originated) [36].

### (e) Sampling proxies and environmental parameters

We extracted a range of sampling proxy and environmental data from the primary literature (section SI 1 in [26]) to test whether extrinsic factors were the drivers of crocodyliform biodiversity dynamics. These parameters can be broadly divided into two categories (see table S3 in section SI 1 in [26]): (i) those that predict biodiversity to be driven by sampling-related artefacts, i.e. non-marine rock outcrop area, and numbers of fossiliferous marine formations [19]; and (ii) those that represent environmental proxies, independent of sampling, i.e. eustatic sea level [38,39]; temperature (*δ*^18^O) using [40], and the independent dataset presented in [11]; the global carbon (*δ*^13^C) and sulfate (*δ*^34^S) cycles [40]; weathering rates (^87^Sr/^86^Sr) [17,40]; as well as an estimate of global subsampled marine invertebrate biodiversity [17], which we use as a coarse proxy for potential food resources for marine crocodyliforms. The residuals of each of these environmental parameters were calculated by using maximum likelihood to fit a first-order autoregressive model, and independently compared using linear regressions to each of our measures of biodiversity. The relative fit of each variable was assessed using the sample-size corrected Akaike information criterion (AICc) and standard correlation tests (see section SI 3 in [26] for detailed protocol).

## 3. Results

### (a) Biodiversity across the J/K boundary

**Figure 2.**
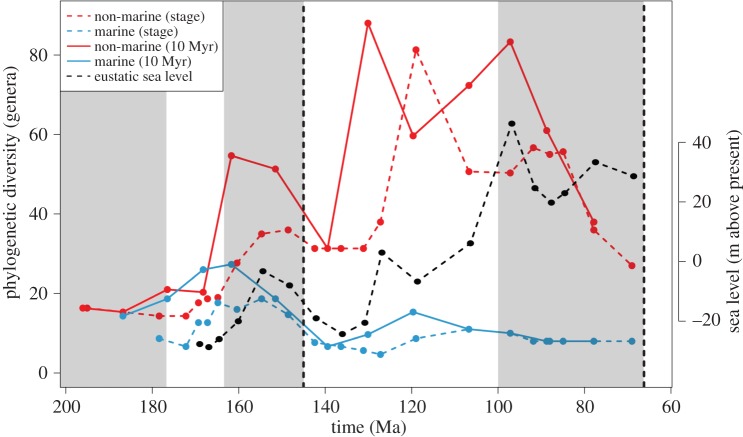
Reconstructed PDE for marine (blue) and non-marine (red) crocodyliforms, based on the mean of all three reconstruction approaches. Eustatic sea level is from Miller *et al*. [38].

**Figure 3.**
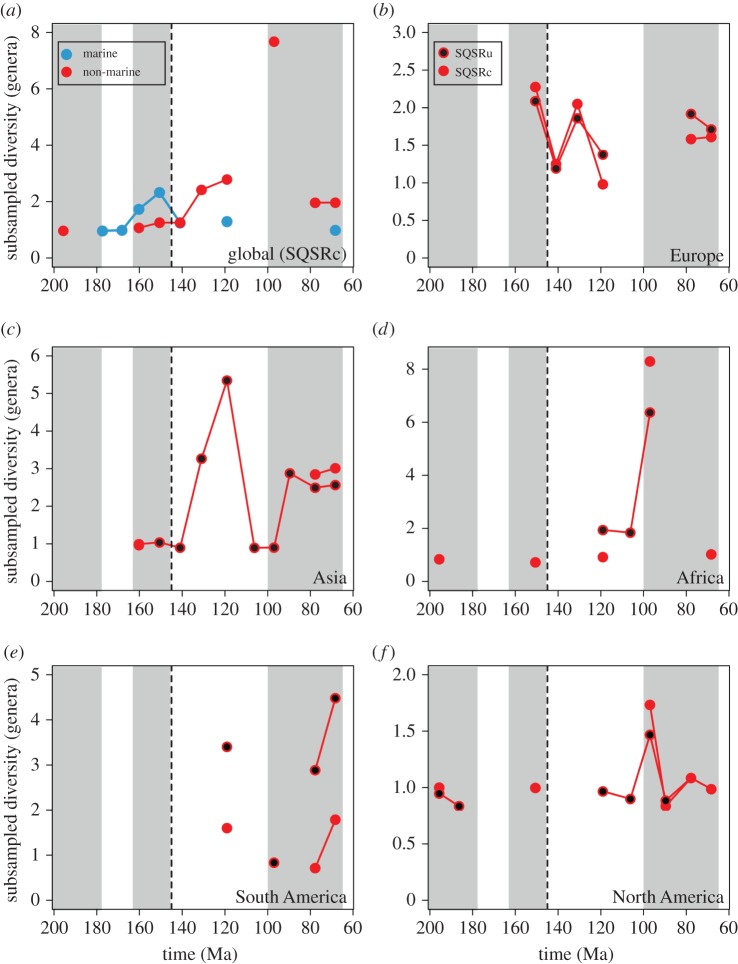
Subsampled biodiversity. (*a*) Marine and non-marine curves (SQSRc); (*b*–*f*) continent-level curves. Red filled circles represent SQSRc, and black filled circles are SQSRu.

An uncorrected (‘raw’) census (TDE) of global non-marine crocodyliform generic biodiversity shows a steady increase from the Middle to Late Jurassic, peaking in the Kimmeridgian–Tithonian, before declining through the J/K boundary (figure 1*c*). Marine biodiversity largely follows this pattern, but there is a much greater biodiversity crash across the J/K boundary (loss of more than 75% genera). Whereas marine biodiversity remained low throughout the Early Cretaceous, non-marine biodiversity partially recovered, but did not reach latest Jurassic levels again during our study interval. This pattern of increasing biodiversity in the Late Jurassic, followed by a sharp decline through the J/K interval, is emulated by our PDE (figure 2) and SQS (figure 3) analyses. PDE and SQSPt are strongly positively correlated with one another for both the marine (Pearson's *r* = 0.601, *p* = 0.115) and non-marine (Pearson's *r* = 0.796, *p* = 0.006) groups.

After the J/K boundary decline, non-marine biodiversity consistently exceeded that of the Late Jurassic based on our PDE, with peaks in the Hauterivian–Barremian and Cenomanian (PDEt), or in the Aptian (PDEs) (figure 2). However, coverage is zero in the Hauterivian–Barremian (i.e. all taxa are known only from singleton occurrences), and we were unable to calculate subsampled biodiversity for this interval. Subsampled results are inconsistent in the non-marine realm: whereas results from the SQSRc analysis show no change in biodiversity through the J/K boundary (figure 3*a*), both the SQSRu (see fig. S2 in section SI 1 in [26]) and SQSPt (see fig. S3 in section SI 1 in [26]) analyses reveal declines of varying strength (57% and 15%, respectively). SQSPs shows a decline in biodiversity from the Tithonian to Berriasian in both the non-marine (54%) and marine realms (45%). The magnitude of the J/K boundary biodiversity decline increases as we raise the quorum level for both marine and non-marine datasets (see fig. S5 in section SI 1 in [26]), suggesting that this is a genuine signal, and not obscured by temporal heterogeneity in sampling intensity. Standard deviations on these biodiversity patterns are consistently low (section SI 4 in [26]), and we estimate the maximum genus extinction level to be around 60–70% for non-marine crocodyliforms, and 75–80% for marine crocodyliforms.

At a palaeocontinental level, poor sampling of earliest Cretaceous (Berriasian–Valanginian) terrestrial deposits generally obscures the spatial dynamics of non-marine crocodyliforms, especially in North America and Gondwana [41]. Within the Laurasian palaeocontinents, latest Jurassic (J6) biodiversity was generally high, but evidence of a decline on land can only be documented in Europe in our SQSPs, SQSRu and SQSRc analyses (39–45% decrease) (figure 3*b*; (see fig. S4 in section SI 1 in [26])). European non-marine biodiversity recovered rapidly in the Hauterivian–Barremian interval, reaching its highest level for any point during the Cretaceous. Based on our results from SQSRu, we are able to show that biodiversity through the J/K boundary in Asia declined only slightly (14% decrease). In Asia (figure 3*c*), Africa (figure 3*d*) and South America (figure 3*e*), Late Jurassic–Early Cretaceous biodiversity peaked in the Aptian (K3), whereas in North America it appears to have been approximately constant (figure 3*f*).

**Figure 4.**
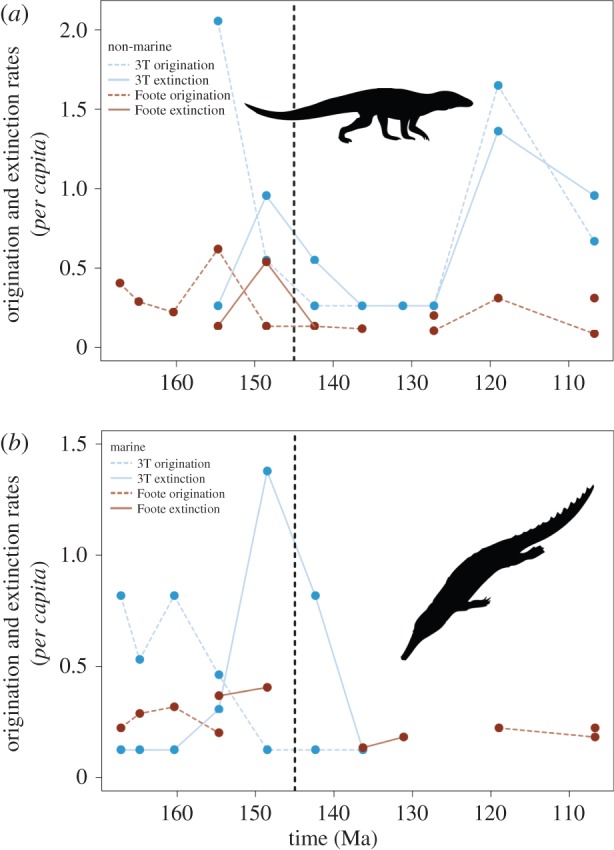
(*a*) Non-marine and (*b*) marine *per capita* extinction rates using the boundary-crosser and three-timer methods.

Following relatively low rates in the Kimmeridgian, both Foote and 3 T extinction rates in non-marine crocodyliforms peaked in the Tithonian (at around four times background rates), remaining high in the Berriasian, before declining through the Valanginian–Barremian (figure 4*a*). Origination rates show a constant pattern of decline in non-marine forms from the Kimmeridgian through the J/K boundary, remaining low throughout most of the Early Cretaceous. Both 3 T origination and extinction rates peaked again in the Aptian. In marine crocodyliforms, the trend is generally similar to that for non-marine crocodyliforms, with the highest extinction rates in the Tithonian and Berriasian (figure 4*b*). However, origination patterns in marine forms are distinct from non-marine crocodyliforms, with very low rates in the Berriasian–Valanginian and no Aptian recovery for marine forms.

### (b) Environmental drivers of biodiversity

A summary of our results that show a strong significant correlation with crocodyliform biodiversity is presented in table 1, with all results documented in S5. TDE shows no strong correlation with any of our extrinsic variables. Sea level is shown to exert the greatest control on marine biodiversity for SQSPs (AICc weight = 0.433), with a significant contribution from *δ*^13^C (AICc weight = 0.259). As we constrained SQSPs to the Bathonian–Albian (see section SI 3 in [26]), these results pertain almost exclusively to thalattosuchians. For SQSRc, no single variable satisfies all of our criteria for statistical significance (see section SI 3 in [26]), but *δ*^34^S and ^87^Sr/^86^Sr isotope cycling are strongly negatively correlated with marine biodiversity (table 1), with some evidence for the importance of sea level too. Although no combination of variables is significantly correlated with SQSPt, it is worth noting that the most important drivers appear to be sea level and palaeotemperature, the latter of which is negatively correlated with biodiversity. Marine PDEt shows a weak and conflicting relationship with sea level, depending on taxonomic scale (section SI 5 in [26]). Contrary to Martin *et al*. [11], we find no positive relationship between marine biodiversity and sea-surface temperature (SST), even when we exclude Metriorhynchoidea (see Discussion).

**Table 1.** Selected results that show strong significant correlations between environmental factors and crocodyliform macroevolutionary dynamics. Full results are provided in section SI 5 in [26].

		AICc		Spearman's correlation		Pearson's correlation	
metric	parameter	likelihood	weight	*ρ*	*p*	*r*	*p*
SQSRc (marine)	*δ*^34^S	19.458	0.240	−0.786	0.048	−0.622	0.136
SQSPt (non-marine)	sea level (Miller)	26.285	0.949	0.750	0.025	0.846	0.004
SQSRu (non-marine)	*δ*^13^C	65.284	0.228	0.762	0.006	0.764	0.004
PDEs (non-marine, genera)	sea level (Miller)	89.704	0.827	0.642	0.033	0.769	0.006
PDEs (non-marine, species)	sea level (Miller)	94.021	0.852	0.873	0.001	0.801	0.003

Changes in eustatic sea level are shown to be the dominant controlling factor on global non-marine crocodyliform biodiversity based on our SQSPt (AICc weight = 0.949) reconstructions of biodiversity (table 1), as well as via our PDEs, with strong statistical support at both the genus and species levels. Sea level is also the strongest driver of non-marine PDEt (AICc weight = 1.0), but this is not supported by our additional correlation tests. SQSRu produces a slightly different association, with a combination of sea level and *δ*^13^C exerting the most control on non-marine biodiversity. Furthermore, analyses for non-marine SQSPs show that there is a strong negative association with SST based on the *δ*^18^O dataset of Martin *et al*. [11] (AICc weight = 0.529).

## 4. Discussion

### (a) Crocodyliform extinction across the J/K boundary

The majority of our results provide strong evidence for a substantial decline in crocodyliform biodiversity across the J/K boundary. This is coupled with high extinction rates in the latest Jurassic (Tithonian), and depressed origination rates throughout the Early Cretaceous (Berriasian–Barremian). The magnitude of this extinction is estimated to have been a loss of approximately 55–75% of total crocodyliform biodiversity at the generic level, with an increase in extinction rate of up to five times that of adjacent time intervals. However, we cannot discount the possibility that at least part of this high extinction rate is due to poor sampling of earliest Cretaceous North American and Gondwanan crocodyliform faunas (see below). These results support those of recent analyses of longer-term trends in marine [5,11,12,19] and non-marine crocodyliform [5] biodiversity, and demonstrate that in spite of high lineage survivability [9,10], there was an overall decline in biodiversity through the J/K boundary. In marine crocodyliforms, this tracks a two-phase thalattosuchian decline, with teleosauroids going extinct at the J/K boundary [9], and metriorhynchoids declining in biodiversity during the Early Cretaceous, prior to their complete extinction by the Aptian [10]. The latter coincides with a steady reduction in the number of thalattosuchian fossil occurrences throughout the Early Cretaceous, despite increasingly better sampling of crocodyliform faunas, providing further support that this was a genuine biodiversity decline.

Even accounting for poor sampling in the earliest Cretaceous, a large biodiversity decrease is still apparent in our PDE reconstructions (figure 2). It has previously been noted that tree instability through errors in phylogenetic tree topology has the effect of ‘dampening’ the magnitude of biodiversity loss, by back-smearing origination times and inflating biodiversity in older time bins [42]. Although this artefact might partially explain heightened biodiversity in the Kimmeridgian–Tithonian, it cannot produce the low biodiversity we recover in subsequent time bins. The J/K biodiversity crash in marine crocodyliforms, and the lack of coverage in the Hauterivian–Barremian, cannot be explained by geological megabias, as other groups of marine reptiles are consistently found in globally distributed deposits throughout this time [12,19]. Therefore, we regard the general lack of marine crocodyliforms in the Hauterivian–Barremian as reflecting a genuine biological signal, rather than a preservation artefact (see also Martin *et al*. [11]). In contrast, non-marine crocodyliform biodiversity recovered rapidly after the J/K boundary, with a peak in the Hauterivian–Aptian that appears to be composed of the radiations of notosuchians and eusuchians [2,3,8], and is a pattern partially mirrored in other terrestrial groups (e.g. dinosaurs [18,43]).

### (b) The impact of sampling on Late Jurassic–Early Cretaceous non-marine crocodyliform biodiversity

The Northern Hemisphere is generally better sampled during the Late Jurassic than its southern counterpart (figure 1*a*,*b*). In Gondwana, we see a sharp reduction in the number of non-marine crocodyliform fossil occurrences across the J/K boundary. This could be due to several different factors: (i) regional crocodyliform extinction, with lineages terminating at the J/K boundary (true absence); (ii) the lack of sedimentary rock availability for sampling fossils (false absence); or (iii) the presence of crocodyliforms, but a failure to sample them among other tetrapod faunas (false absence). In North America, the earliest Cretaceous (Berriasian–Barremian) is largely devoid of tetrapod fossils [41] (section SI 4 in [26]), and therefore we can infer that the lack of crocodyliforms is most likely the product of poor sampling. In Europe, the continental Berriasian record is relatively well sampled, but still documents a decline in non-marine crocodyliform biodiversity (figure 3*b*; section SI 4 in [26]). This European decline is tracked by a constriction in the apparent latitudinal ranges of Northern Hemisphere earliest Cretaceous crocodyliforms across the J/K boundary (see fig. S1 in section SI 1 in [26]). In Asia, the first well-dated Cretaceous occurrences are from the Hauterivian–Barremian of the Russian Federation (section SI 2 in [26]), and the low Berriasian–Valanginian biodiversity (SQSRu) we find is based on rare semi-aquatic occurrences from poorly temporally constrained localities. Other small-bodied groups, such as lepidosaurs and mammals, are also rare in earliest Cretaceous Asian faunas, whereas dinosaur fossils are relatively well known [41], although these groups all occupied different non-marine environments in Asia throughout this time and have variable preservational potentials [41]. Despite these differences, the rarity of crocodyliform fossils suggests that at least a portion of the low biodiversity of this group in the earliest Cretaceous is a genuine signal, but we cannot rule out that part of this is due to incomplete sampling.

In Africa, the first identifiable Cretaceous crocodyliform occurrences are from the Aptian, represented by the notosuchians *Malawisuchus* and *Araripesuchus* from Malawi [44]. In South America, the earliest Cretaceous record is restricted to just a single occurrence of the Brazilian neosuchian *Susisuchus*, which cannot be dated more precisely than the Berriasian–Barremian [45]. However, there are relatively high numbers of dinosaur-bearing collections and formations in the earliest Cretaceous of Gondwana [41], including regions inhabited by crocodyliforms during other intervals of the Mesozoic. Therefore, the absence of non-marine crocodyliforms from these regions at this time cannot be fully explained by sampling failure and reflects at least in part a genuine lack of biodiversity, a pattern also observed in contemporaneous Gondwanan turtle faunas [46].

### (c) Environmental drivers of the J/K crocodyliform biodiversity crash

Our corrected biodiversity curves are largely convergent and show varying degrees of correlation with a range of environmental factors (table 1; section SI 5 in [26]), in contrast to raw taxonomic biodiversity. This suggests that our methods of reconstructing biodiversity are appropriate, and do not remove an underlying sampling or biodiversity signal.

After correcting for sampling, we were unable to recover the positive relationship between episodes of warm SST and marine crocodyliform biodiversity found by Martin *et al*. [11]. Our lack of correlation occurs despite using the same SST dataset and a similar phylogenetic correction methodology to those authors. Furthermore, no relationship was recovered for our subsampled results based on SQSPs and SQSPt, and our SQSRc analysis actually produced a statistically weak negative correlation between SST and marine biodiversity (section SI 5 in [26]). This disagreement could be due to the different statistical procedure employed by Martin *et al*. [11], as well as the relatively short temporal duration of thalattosuchians (an issue that is alleviated by our use of a maximum-likelihood modelling approach). However, this discrepancy more probably pertains to the treatment of metriorhynchoid thalattosuchians. Martin *et al*. [11] only recovered a positive correlation between biodiversity and SST when they excluded metriorhynchoids. These authors suggested that this group responded differently to palaeotemperature changes than other marine crocodyliforms. However, a simpler explanation is that there is no strong palaeotemperature signal governing the long-term trends in marine crocodyliform biodiversity [5]. When we exclude metriorhynchoids from our analyses using SQSPs, we find that Late Jurassic teleosauroid diversity remains flat until their extinction at the J/K boundary (section SI 4 in [26]), and we are still unable to recover a positive relationship with palaeotemperature (Pearson's *r* = −0.69, *p* = 0.197). If metriorhynchoids are excluded from our PDE analyses, a weak positive association is recovered between marine biodiversity (PDEs) and palaeotemperature (Spearman's *ρ* = 0.524, *p* = 0.098), but our AICc results support a stronger relationship with *δ*^34^S (AICc weight = 0.283). Furthermore, the relationship between PDEt and sea level is strengthened when metriorhynchoids are excluded at both the genus (AICc weight = 0.873) and species (AICc weight = 0.998) levels. Overall, our results support those of Mannion *et al*. [5] in that eustatic sea level was the most important factor in controlling the biodiversity of marine crocodyliforms. This correlation is most strongly recovered for PDEt and SQSPs, and periods of high biological activity in the oceans (indicated by *δ*^13^C) also appear to be a strong controlling factor for SQSPs. While some of our analyses do not fully support this relationship with sea level (SQSPt, SQSRc, PDEs), these results are non-significant and do not necessarily contradict our conclusions. Our results for SQSRc also suggest that factors such as nutrient cycling and eustacy-influenced redox shifts (indicated by perturbations to the *δ*^34^S cycle) were also important in regulating marine crocodyliform biodiversity, as secondary mechanisms underpinned by fluctuating sea levels.

Interestingly, our results also indicate that sea level influenced non-marine crocodyliform biodiversity. Rising sea levels increase the amount of shallow marine habitat available, resulting in high biodiversity during the Late Jurassic highstand. Sea level reached a global lowstand across the J/K boundary [38,47,48], reflected in a reduction of global crocodyliform biodiversity. Because most of the Late Jurassic crocodyliforms in our non-marine dataset are coastal or semi-aquatic forms (e.g. Atoposauridae, Goniopholididae), rather than fully terrestrial (e.g. Notosuchia), it seems likely that these major eustatic sea-level changes promoted high Late Jurassic biodiversity, as well as the elevated extinctions and subsequent low biodiversity of crocodyliforms in both the marine and non-marine realms. This conclusion should be treated with caution because much of this non-marine signal might be a reflection of changes in European basins across the J/K boundary. Nevertheless, our non-marine results are consistent with the conclusions of a range of studies on vertebrates [19] and invertebrates [17,49–51], that suggest eustatic sea-level changes exhibit a first-order control on the evolution of near shore ecosystems.

The Early Cretaceous witnessed a series of ‘biocalcification crises’ (e.g. in the Valanginian and Aptian) that saw a dramatic reduction in the production of carbonates [52,53], and potentially decreased the amount of habitable areas for shallow-marine dwelling crocodyliforms. Furthermore, there is evidence that the global drop in eustatic sea level at the J/K boundary decimated reef environments [54,55], and there were elevated extinction rates for sessile groups of cephalopods, bivalves and gastropods at low palaeolatitudes [20,32,56]. These events culminated in several episodes of intense ocean water stagnation and anoxia, including the Valanginian Weissert carbon isotope excursion and the late Hauterivian Faraoni oceanic anoxic events [52,53,57]. It is likely that these environmental events played a prominent role in our recovery of a strong positive association between fluctuations in biodiversity and sea level, as well as the strong correlation between SQSRu and *δ*^13^C, and potentially provided the *coup de grâce* for Thalattosuchia. This indicates that large-scale tectonic processes, relating to the ongoing fragmentation of Pangaea, were influential in shaping crocodyliform biodiversity dynamics through the J/K boundary, by increasing rates of continental weathering and the heightened influx of inorganic nutrients into marine basins [58,59].

### (d) Ecological implications of a crocodyliform Jurassic/Cretaceous biodiversity crash

Along with the decline and final extinction of marine thalattosuchian crocodylomorphs in the Early Cretaceous, multiple non-marine turtle groups (e.g. basal eucryptodirans, eurysternids and plesiochelyids) disappeared in Europe at the J/K boundary [60]. This might have been important in releasing ecological pressure, resulting in opportunistic replacement by marine macropredaceous groups, such as the diversification of plesiosaurian [14] and shark [61] lineages immediately after the J/K boundary, and the subsequent diversification of pancryptodiran and pleurodiran turtles [62,63]. The radiation of these clades suggests that there might have been broader ecological shifts occurring in semi-aquatic to shallow marine reptile faunas [64], and the occupation of high tier predatory niches by new groups was likely an important factor in suppressing the recovery of marine crocodyliforms. This pattern is distinct from that observed in continental crocodyliform ecosystems: there we see a drop in biodiversity followed by a rapid recovery and subsequent radiations (Eusuchia and Notosuchia) during the Early Cretaceous [2,8], representing a faunal turnover in non-marine crocodyliform faunas as ecological pressure was released following the J/K boundary decline. Therefore, although we have identified several key environmental drivers of crocodyliform biodiversity dynamics through the J/K boundary, we cannot reject the possibility that a combination of ecological aspects also influenced crocodyliform evolutionary patterns during this interval.

## 5. Conclusion

Using a combined approach to reconstructing palaeobiodiversity, we have demonstrated that crocodyliforms suffered a major biodiversity decline across the Jurassic/Cretaceous boundary in both the marine and terrestrial realms. This is accompanied by elevated extinction rates in the latest Jurassic, nearly at the level of mass extinction status, and severely depressed origination rates in the Early Cretaceous. Sea-level changes were primarily responsible for this biodiversity decline, both in the marine realm and on land, reducing the amount of habitable shallow marine area for crocodyliforms. Secondary factors driving biodiversity changes included perturbations to the carbon and sulfur cycles that, together with sea-level fluctuations, indicate a prominent role for large-scale tectonic processes in shaping crocodyliform biodiversity in the Late Jurassic to Early Cretaceous. Contrary to previous work, we find little evidence for a mediating effect of palaeotemperature on crocodyliform biodiversity during this interval. Overall, this suggests that the fate of Mesozoic crocodyliforms was coupled more broadly to a combination of environmental factors and their wider impact on pelagic and shallow marine ecosystems. Our results support the hypothesis that sea-level change is the principal driving factor in shaping the evolution of shelf biotas, but we cannot rule out that additional ecological factors were also at play across the Jurassic/Cretaceous boundary.

## Note added in proof

After acceptance of our paper, two papers were published relevant to our study. One [65] argued that the stratigraphically youngest thalattosuchian [10] might instead be a brachauchenine pliosaurid. The other [66] described a single, highly-specialised Early Cretaceous (Hauterivian) southern Tethyan representative of Teleosauridae from the, a group previously thought to have gone extinct at the J/K boundary. Although these potentially change the timings of extinctions in the marine realm, they do not have a notable impact on our analyses or results: the former is an indeterminate occurrence (cf. Plesiosuchina indet.) that is not included in our analyses; and the latter requires that only a single additional lineage passed through the J/K boundary, and also as a singleton occurrence would not have been included in our subsampling trials.

## Data accessibility

All supplementary material has been included as part of the associated Dryad package with this manuscript: http://datadryad.org/handle/10255/dryad.98760.
